# Notifications of sexual violence against children and adolescents in Rio Grande do Sul, Brazil: a descriptive study, 2014-2018

**DOI:** 10.1590/S2237-96222023000200004

**Published:** 2023-07-14

**Authors:** Samara da Silveira Lourenço, Maurício Polidoro, Luciane Maria Piloto, Aline Blaya Martins

**Affiliations:** 1 Universidade Federal do Rio Grande do Sul, Programa de Pós-Graduação em Saúde Coletiva, Porto Alegre, RS, Brazil; 2 Instituto Federal do Rio Grande do Sul, Porto Alegre, RS, Brazil

**Keywords:** Child Abuse, Sexual, Sex Offenses, Racism, Mandatory Reporting, Health Information Systems, Epidemiology, Descriptive, Abuso Sexual Infantil, Delitos Sexuales, Racismo, Notificación Obligatoria, Sistemas de Información en Salud, Epidemiología Descriptiva, Abuso Sexual de Crianças e Adolescentes, Delitos Sexuais, Preconceito Racial, Notificação de Abuso, Sistemas de Informação em Saúde, Epidemiologia Descritiva

## Abstract

**Objective:**

to describe characteristics of notifications of sexual violence against children and adolescents according to race/skin color and their distribution in the state of Rio Grande do Sul, Brazil, between 2014 and 2018.

**Methods:**

this was a descriptive study of data retrieved from the Notifiable Health Conditions Information System (SINAN). Frequency distributions, prevalence and statistical differences were analyzed using Pearson’s chi-square test.

**Results:**

of the 8,716 notifications, most occurred in the state capital (48.2%) and related to female victims (82.2%) aged between 10 and 14 years (38.1%). There was a higher prevalence (370/100,000) and relative frequency of rape (84.5%), sexual exploitation (5.8%) and neglect/abandonment (4.6%) among victims of Black race/skin color (p-value < 0.05). Only 4.6% of notifications occurred in primary health care services.

**Conclusion:**

notifications were more frequent among female pre-adolescents and prevalence was higher among Black people, who should be a priority target for protective measures. Surveillance of this form of violence needs to be strengthened in primary care.

## Resumo

**Objetivo:**

descrever características das notificações de violência sexual contra crianças e adolescentes segundo a raça/cor da pele e sua distribuição no Rio Grande do Sul, Brasil, 2014-2018.

**Métodos:**

estudo descritivo de dados do Sistema de Informação de Agravos de Notificação (Sinan); foram analisadas distribuições de frequência, prevalências e diferenças estatísticas pelo teste qui-quadrado de Pearson.

**Resultados:**

das 8.716 notificações, a maioria ocorreu na capital (48,2%) e de vítimas do sexo feminino (82,2%) com idade entre 10 e 14 anos (38,1%); houve maior prevalência (370/100 mil) e frequência relativa de estupro (84,5%), exploração sexual (5,8%) e negligência/abandono (4,6%) entre vítimas de raça/cor da pele negra (p-valor < 0,05); 4,6% das notificações foram realizadas nos serviços de Atenção Básica à Saúde (ABS).

**Conclusão:**

houve maior frequência de notificações entre pré-adolescentes do sexo feminino e maior prevalência entre negros, alvos prioritários das medidas de proteção; torna-se necessário reforçar a vigilância dessa violência na ABS.

## Resumen

**Objetivo:**

describir las características de las notificaciones de violencia sexual contra niños y adolescentes según raza/color de piel y su distribución en Rio Grande do Sul, Brasil, 2014-2018.

**Métodos:**

estudio descriptivo de datos del Sistema de Información de Enfermedades y Notificaciones (Sinan). Distribuciones de frecuencia, prevalencias y diferencias estadísticas fueron analizadas mediante la prueba de chi-cuadrado de Pearson.

**Resultados:**

de 8.716 notificaciones, la mayoría ocurrió en la capital (48,2%) y fueron de víctimas del sexo femenino (82,2%) con edades entre 10 y 14 años (38,1%). Hubo mayor prevalencia (370/100.000) y frecuencia relativa de violación (84,5%), explotación sexual (5,8%) y negligencia/abandono (4,6%) entre víctimas de raza/color de piel negro (p-valor < 0,05). Un 4,6% de las notificaciones ocurrió en servicios de atención primaria.

**Conclusión:**

hubo mayor frecuencia de notificaciones entre preadolescentes femeninas y mayor prevalencia entre víctimas negras, que deben presentar medidas de protección prioritarias. Es necesario reforzar la vigilancia de violencias en atención primaria.

**Table:** 

Study contributions	
**Main results**	Most notifications related to female pre-adolescents; there was higher prevalence and relative frequency of rape, sexual exploitation and neglect among Black victims; notifications made by hospitals in the state capital were predominant.
**Implications for services**	The results of the study show low surveillance of sexual violence by Primary Health Care services in the state, especially in municipalities with higher rates of violence, hindering identification of cases and the problem being addressed.
**Perspectives**	Public policies aimed at addressing violence require principles of equity; future studies analyzing intersectionality between race/skin color, gender and social class in sexual violence are important for informing such actions.

## INTRODUCTION

Prevalence of sexual violence against children and adolescents is high in Brazil and there are many challenges to be addressed.^1^ Besides affecting prematurely the multidimensionality of victims, which includes physical, reproductive and mental health, this form of violence, which has multiple consequences in society,^2^ is defined by the World Health Organization (WHO) as:

The involvement of a child or an adolescent in sexual activity that he or she does not fully comprehend and is unable to give informed consent to, or for which the child or adolescent is not developmentally prepared.^3^

In Brazil sexual violence is classified according to the following categories: (i) sexual harassment, defined as inappropriate insistence, regardless of sex or sexual orientation, with questions, proposals, pretensions or any other form of forced approach of a sexual nature; (ii) rape, which consists of compelling someone, by means of violence or serious threat, to have sexual intercourse or to practice or allow other libidinous acts to be practiced with them; (ii) child pornography, which includes the presentation, production, sale, supply, dissemination and/or publication of photographs or images with pornography or explicit sex scenes involving children or adolescents, using any means of communication; and (iv) sexual exploitation, characterized by the use of people, regardless of age, sex or gender identity, for commercial and profit purposes, whether for the practice of prostitution, or for exposing naked bodies and sexual intercourse, either live or through published images.^4^

Cases of sexual violence against children and adolescents are often underreported by the authorities responsible for this, mainly due to silencing of victims, motivated both by fear of the aggressor and by fear of not being believed by family members.^5^ Therefore, health professionals need to be trained and attentive in order to identify signs of violence, provide the necessary assistance to victims and their families and report the case to the competent authorities.

The Child and Adolescent Statute (*Estatuto da Criança e do Adolescente* - ECA), enacted in 1990, guarantees protection from all forms of negligence, discrimination, exploitation, violence, cruelty and oppression, and in this sense, this law made it mandatory to notify suspected or confirmed cases of abuse against children and adolescents.^6^ The Violence and Accident Surveillance System (*Sistema de Vigilância de Violências e Acidentes* - VIVA), created in 2006, was the first initiative to systematize data on violence in the country. As of 2009, the component related to the surveillance of cases of domestic, sexual and/or other interpersonal and self-inflicted violence was integrated into the Notifiable Diseases Information System (*Sistema de Informação de Agravos de Notificação* - SINAN), already consolidated nationwide at that time.^4^

In Brazil, there is still need for more in-depth knowledge about data on sexual violence against children and adolescents, especially at the regional level.^7^ Furthermore, there is a scarcity of studies dedicated to investigating this form of violence according to race/skin color in Brazil.^8^ Therefore, in order to monitor notifications of sexual violence against children and adolescents in states and municipalities, as well as to identify its characteristics according to the victims’ race/skin color, the need exists to propose decentralized strategies to address it.

The objective of this study was to describe characteristics of notifications of sexual violence against children and adolescents according to race/skin color and their distribution in the state of Rio Grande do Sul, Brazil, between 2014 and 2018.

## METHODS

*Design*

This was a descriptive cross-sectional study of cases of sexual violence against children and adolescents reported on the SINAN.

*Background*

The state of Rio Grande do Sul consists of 497 municipalities and has a population of 10,693,929 inhabitants, according to the most recent demographic census, carried out by the Brazilian Institute of Geography and Statistics (*Instituto Brasileiro de Geografia e Estatística* - IBGE) in 2010. In that year, 3,105,241 children and adolescents accounted for 29% of the state’s total population.^9^ Currently, with regard to race/skin color, the population of Rio Grande do Sul self-reports itself as 83.2% White, 10.6% mixed race, 5.5% Black, 0.3% Asian and 0.3% Indigenous. Specifically, with regard to children and adolescents, 2,583,560 are White and 499,944 are mixed race and Black.^10^

SINAN data are published in the state by the Rio Grande do Sul State Health Surveillance Center (*Centro Estadual de Vigilância em Saúde* - CEVS/RS), which is the service responsible for developing state health surveillance policies. The competencies and responsibilities of the CEVS/RS were defined by Decree 44.050, dated October 5, 2005.^11^ The SINAN uses an Individual Notification Form (*Ficha de Notificação Individual* - FNI), available in more detail on the following website: http://portalsinan.saude.gov .br/notificacoes. The FNI is composed of variables, grouped together in blocks, which were collected and reorganized for the purpose of our analysis.

The notifications in question must be made by any establishment that perceives a confirmed or suspected case of violence, such as health centers, social services or schools. The municipal health centers have the task of collecting the FNIs and forwarding them, on an ongoing basis, to the municipal Health Departments. These are in charge of inputting the data to the system and forwarding it to the state Health Departments and the Ministry of Health.^4^

*Participants*

Notifications of sexual violence perpetrated against children, aged between 0 and 9 years, and adolescents, between 10 and 19 years old, were considered eligible for the study. Those aged between 10 and 14 years were categorized as pre-adolescents, as defined by the WHO and which the Health Ministry adopts in its health policies.^12^

*Variables*

We analyzed variables related to the characteristics of victims, aggressors and occurrences of violence, as well as the distribution of notifications:

a) Characteristics of the victims

- age group (at last birthday: 0-5; 6-9; 10-14; 15-19);

- sex (female; male);

- race/skin color [White; Black (the “Black” and “mixed-race” categories grouped together)]; and

- disability (physical; intellectual; visual; hearing) or disorder (mental; behavior) (yes; no);

b) Characteristics of the aggressors

- sex (male; female);

- relationship to victim [father/mother; stepfather/stepmother; blood relative; non-blood relative; spouse/partner/ex-spouse/ex-partner; friend/acquaintance; school/carer; stranger; other (person with an institutional relationship; boss; police officer/law enforcement officer; other)] - the “other” category required mandatory specification, in writing, and was able to be analyzed separately; some of these data were grouped into categories already defined on the FNI, while others generated new categories, such as “blood relative” [which, in addition to grouping the sibling and child categories, included data related to cousins, uncles/aunts, grandparents , great-uncles/aunts and great-grandparents], “school/carer” [which grouped the together in the “caregiver” category data related to teachers, school monitors, school/day care colleagues, nannies, secretaries, housekeepers, bus drivers, spouses and other relatives of caregivers] and “non-blood relative” [which grouped the data related to spouses or partners of grandparents, parents of stepfathers/stepmothers, brothers/sisters-in-law, in-laws, godparents, partners of parents and relatives of stepfathers/stepmothers];

- life cycle of the victim [in years: child (0-9); adolescent (10-19); young adult (20-24); adult (25-59); and elderly (60 or over); and

- Number of people involved (1; 2 or more);

c) Characteristics of the occurrences

- place [home; school; public thoroughfare; other (collective housing; sports facility; bar or similar; trade/services; industries/construction; or any other place)];

- recurrence (yes; no);

- type (sexual harassment; rape; sexual exploitation; child pornography; other) - the “other” category was answered in full and, after analyzing each notification, one by one, some of the data were grouped into the four other categories; prior to 2015, the FNI had a fifth category, “violent indecent assault” (atentado violento ao pudor - AVP), which, after the enactment of Law No. 12.015, dated August 7, 2009, became the crime of rape;^13^ as such, notifications in the AVP category were grouped into the rape category; and

- co-occurrence (other forms of violence: physical violence; psychological/moral violence; neglect/abandonment);

d) Distribution of the notifications

- municipality of notification (all the municipalities of the state); and

- type of notifying service [hospital services; Primary Health Care services (primary health care centers and Family Health Strategy centers); emergency health services; medical outpatient and polyclinic services; medical specialty services; social services; others].

*Data source and measurement*

We used secondary data from the SINAN, provided by the CEVS/RS, extracted in April 2020 (for the period from 2014 to 2017) and in July 2022 (for 2018). The data made available showed all types of violence against women and men, of all ages. For the purposes of this study, first of all we separated data on sexual violence and then the data in this category relating only to individuals under 19 years old.

In order to calculate prevalence rates, we used IBGE population data derived from the 2010 Census.^9^ ^),(^ ^10^

*Statistical method*s

The frequency distribution of the characteristics of victims, aggressors and occurrences of violence was analyzed according to the race/skin color of the victims (White; Black). Differences between groups were analyzed using Pearson’s chi-square test. The prevalence of notifications of sexual violence against children and adolescents was obtained as follows: the total number of notifications in the five-year period analyzed, 2014-2017, was divided by the total number of children and adolescents residing in the state (per 100,000). We also calculated notification prevalence, disaggregated by race/skin color, dividing the number of notifications of White or Black victims, referring to the period 2014-2017, by the population of White and Black children and adolescents residing in the state (per 100,000), with a correction factor of 1.04. We chose not to include notifications relating to Asian or Indigenous race/skin color in the analysis, given their low prevalence in the state (each one accounts for 0.3% of the total population)^10^ and the low number of such notifications in the study period.

We described the percentage distribution of notifications by municipality and by type of notifying services. We also calculated notification prevalence according to major municipality (> 100,000 inhabitants), as follows:^14^ the number of notifications of sexual violence against children and adolescents for each municipality, in the five-year period analyzed, was divided by the number of children and adolescents residing in the municipality (per 100,000). We built a map showing the distribution of the number of notifications (0; 1-49; 50-203; 204-510; 511-4,201), by municipality, using TabWin version 3.6b. We analyzed the data using the R open source program, version 4.1.3, RStudio interface version 1.3.1093.

*Ethical aspects*

The research project was approved by the Escola de Saúde Pública Health Research Ethics Committee, an institution linked to the Rio Grande do Sul State Health Department, as per Opinion No. 5.497.842, issued on July 29, 2022, and Certificate of Submission for Ethical Appraisal (*Certificado de Apresentação para Apreciação Ética* - CAAE) No. 69992817.5.3001.5312. It was also approved by the *Universidade Federal do Rio Grande do Sul* Teaching and Research Center, as per Opinion No. 3.999.164, issued on April 19, 2020 (CAAE No. 69992817.5.0000.5347).

## RESULTS

Between 2014 and 2018, 11,099 cases of sexual violence were notified in Rio Grande do Sul; these included 3,965 (35.7%) victimized children and 4,751 (42.8%) adolescents, totaling 8,716 (78.5%) cases, which represents a prevalence of 281 notifications of sexual violence per 100,000 children and adolescents residing in the state. Most notifications of sexual violence against these children and adolescents related to victims of White race/skin color (78.5%) (Table 1). The prevalence rate of notifications (370/100,000) for Black victims was higher than that found for White victims (262/100,000).

**Table 1:** Characteristics of victims of sexual violence against children and adolescents notified on the Notifiable Health Conditions Information System (n = 8,284), by race/skin color, Rio Grande do Sul, 2014-2018

Variables	Race/skin color		Total n (%)	p-value^a^
	White	Black		
	n (%)	n (%)		
	6,503 (78.5)	1,781 (21.5)	8,284 (100.0)	
Age group (at last birthday)				
0-5	1,544 (23.7)	324 (18.2)	1,868 (22.6)	< 0.001
6-9	1,524 (23.4)	359 (20.2)	1,883 (22.7)	
10-14	2,390 (36.8)	765 (42.9)	3,155 (38.1)	
15-19	1,045 (16.1)	333 (18.7)	1,378 (16.6)	
Sex^b^				
Female	5,372 (82.6)	1,437 (80.7)	6,809 (82.2)	0.060
Male	1,130 (17.4)	344 (19.3)	1,474 (17.8)	
Disability or disorder^c^				
Yes	558 (9.3)	193 (11.9)	751 (9.9)	0.002
No	5,420 (90.7)	1,425 (88.1)	6,845 (90.1)	

a) Pearson’s chi-square test; b) 1 (one) data item missing (unknown) for this variable (n = 8,283); c) 688 records with unknown data or left blank (n = 7,596).

Notification frequency was higher in the 10-14 age group, and among victims of Black race/skin color, for whom frequency was 42.9%; while among victims of White race/skin it was 36.8%. There was a predominance of female victims (82.2%), in both race/skin color groups. 9.3% and 11.9% of victims of White and Black race/skin color, respectively, had a disability or disorder (p-value = 0.002) (Table 1).

With regard to the aggressors, the majority were male (94.5%), adults (59.3%) and friends/acquaintances of the victims (27.4%), blood relatives (18.2%), father/mother (16.8%) or stepfather/stepmother (14.5%), with no significant differences in relation to the victims’ race/skin color. There was a higher frequency of notifications involving two or more aggressors among victims of Black race/skin color (17.7%) when compared to those of White race/skin color (14.6%; p-value < 0.001) (Table 2).

**Table 2:** Characteristics of victims of sexual violence against children and adolescents notified on the Notifiable Health Conditions Information System (n = 8,284), by race/skin color, Rio Grande do Sul, 2014-2018

Variables	Race/skin color		Total n (%)	p-value^a^
	White	Black		
	n (%)	n (%)		
Sex^b^				
Male	5,937 (94.7)	1,611 (93.7)	7,548 (94.5)	0.100
Female	146 (2.3)	41 (2.4)	187 (2.3)	
Both sexes^c^	184 (3.0)	68 (3.9)	252 (3.2)	
Relationship to victim^d^				
Father/mother	1,077 (17.5)	244 (14.6)	1,321 (16.8)	0.100
Stepfather/stepmother	901 (14.6)	236 (14.1)	1,137 (14.5)	
Blood relative	1,117 (18.1)	306 (18.3)	1,423 (18.2)	
Non-blood relative	250 (4.1)	66 (4.0)	316 (4.0)	
Spouse/partner/ex-spouse/ex-partner	246 (4.0)	72 (4.3)	318 (4.1)	
Friend/acquaintance	1,669 (27.0)	478 (28.6)	2,147 (27.4)	
School/carer	100 (1.6)	20 (1.2)	120 (1.5)	
Stranger	618 (10.0)	190 (11.4)	808 (10.3)	
Other	189 (3.1)	59 (3.5)	248 (3.2)	
Life cycle^e^				
Child	110 (2.4)	24 (2.0)	134 (2.3)	0.040
Adolescent	944 (20.3)	277 (22.8)	1,221 (20.8)	
Young adult	522 (11.2)	159 (13.0)	681 (11.6)	
Adult	2,788 (59.8)	696 (57.2)	3,484 (59.3)	
Elderly	294 (6.3)	61 (5.0)	355 (6.0)	
Number of people involved^f^				
1	5,241 (85.4)	1,382 (82.3)	6,623 (84.7)	< 0.001
≥ 2	895 (14.6)	298 (17.7)	1,193 (15.3)	

a) Pearson’s chi-square test; b) 297 missing data for this variable, due to unknown data or fields left blank (n = 7,987); c) Variable relating to violence perpetrated by two or more aggressors of different sexes; d) 446 missing data for this variable due to unknown data or fields left blank (n = 7,838); e) 2,446 missing data for this variable (unknown data or left blank) (n = 5,838); f) 468 missing data for this variable (unknown data or left blank) (n = 7,816).

As for the place of occurrence, the home of the victims was predominant (76.6%), and this was not related to race/skin color. Recurrence of sexual violence, that is, when the violence was perpetrated more than once against the same victim, accounted for more than half of the cases (60.2%), among children and adolescents of both races/skin colors (Table 3).

**Table 3:** Characteristics of occurrences of sexual violence against children and adolescents notified on the Notifiable Health Conditions Information System (n = 8,284), according to victims’ race/skin color, Rio Grande do Sul, 2014-2018

Variables	Race/skin color		Total n (%)	p-value^a^
	White	Black		
	n (%)	n (%)		
Place^b^				
Home	4,674 (76.9)	1,264 (75.5)	5,938 (76.6)	0.400
School	155 (2.6)	36 (2.2)	191 (2.5)	
Public thoroughfare	507 (8.3)	154 (9.2)	661 (8.5)	
Other	742 (12.2)	220 (13.1)	962 (12.4)	
Recurrence^c^				
Yes	3,063 (60.1)	853 (60.8)	3,916 (60.2)	0.600
No	2,036 (39.9)	551 (39.2)	2,587 (39.8)	
Type ^d^				
Sexual harassment^e^	1,709 (27.7)	428 (25.4)	2,137 (27.2)	0.049
Rape^f^	5,064 (82.5)	1,428 (84.5)	6,492 (82.9)	0.040
Child pornography^g^	173 (2.9)	46 (2.8)	219 (2.8)	0.900
Sexual exploitation^h^	231 (3.8)	96 (5.8)	327 (4.3)	< 0.001
Outro^i^	426 (7.2)	97 (6.0)	523 (6.9)	0.090
Co-occurrence^d^				
Physical^j^	991 (15.6)	280 (16.2)	1,271 (15.8)	0.600
Psychological/moral^k^	1,912 (30.0)	552 (31.9)	2,464 (30.4)	0.100
Neglect/abandonment^l^	215 (3.4)	80 (4.6)	295 (3.6)	0.010

a) Pearson’s chi-square test; b) 532 missing data for this variable (unknown; left blank) (n = 7,752); c) 1,781 missing data for this variable (unknown; left blank) (n = 6,503); d) More than one category is allowed for this variable; e) 440 missing data for this variable (unknown; not applicable; left blank) (n = 7,844); f) 454 missing data for this variable (unknown; not applicable; left blank) (n = 7,830); g) 645 missing data for this variable (unknown; not applicable; left blank (n = 7,639); h) 522 missing data for this variable (unknown; not applicable; left blank (n = 7,762); i) 711 missing data for this variable (unknown; not applicable; left blank) (n = 7,573); j) 215 missing data for this variable (unknown; left blank) (n = 8,069); k) 184 missing data for this variable (unknown; left blank) (n = 8,100); l) 150 missing data for this variable (unknown; left blank) (n = 8,134).

The most frequent type of violence was rape (82.9%), followed by sexual harassment (27.2%). The relative frequencies of rape and sexual exploitation were higher among victims of Black race/skin color (84.5% and 5.8%, respectively), when compared to victims of White race/skin color (82.5% and 3.8%, respectively), while sexual harassment was more frequent among victims of White race/skin color, compared to those of Black race/skin color (27.7% versus 25.4%; p-value = 0.049 ). The main co-occurrences were psychological/moral violence (30.4%) and physical violence (15.8%). Co-occurrence of neglect/abandonment was higher among victims of Black race/skin color (4.6%) when compared to those of White race/skin color (3.4%; p-value = 0.010) (Table 3).

**Figure 1:**
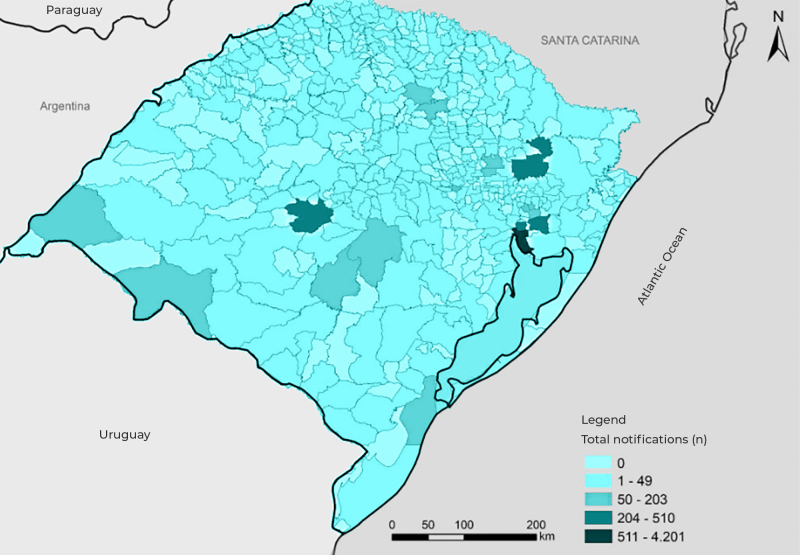
Distribution of notifications of sexual violence against children and adolescents (n = 8,716), by municipality, Rio Grande do Sul, 2014-2018

The city of Porto Alegre, the state capital, had the highest number of cases (4,201 notifications; 48.2%), followed by Caxias do Sul (510; 5.8%), Gravataí (345; 4.0%), Canoas (336; 3.8%) and Santa Maria (278; 3.2%). Of the state’s 497 municipalities, 291 (58.5%) had less than 50 notifications and 189 (38.0%) had no notifications, over the five years studied (Box 1; Figure 1). Among the major municipalities, Alvorada (0.1%), Cachoeirinha (0.3%), Bagé (0.3%), Sapucaia do Sul (0.3%) and Pelotas (0.4%) were those with the lowest number of notifications. When calculating the prevalence of notifications made per municipality, in the five-year period analyzed, Alvorada and Pelotas had the lowest numbers, 11 and 35 per 100,000 children and adolescents, respectively (Box 1).

**Box 1:** Prevalence of notifications of sexual violence against children and adolescents, by major municipality, Rio Grande do Sul, 2014-2018

Major municipalities^a^	Number of children and adolescents^b^	Number of notifications	Prevalence of notifications (per 100,000 children and adolescents)
Porto Alegre	367,306	4,201	1,144
Caxias do Sul	121,620	510	419
Pelotas	91,776	32	35
Canoas	99,058	336	339
Santa Maria	73,311	278	379
Gravataí	80,283	345	430
Viamão	79,530	39	49
Novo Hamburgo	71,208	70	98
São Leopoldo	66,274	49	74
Rio Grande	58,086	203	349
Alvorada	69,772	8	11
Passo Fundo	55,165	150	272
Sapucaia do Sul	41,185	30	73
Uruguaiana	43,841	108	246
Santa Cruz do Sul	31,645	39	123
Cachoeirinha	35,623	23	65
Bagé	35,502	26	73
Bento Gonçalves	27,157	77	284

a) For the purpose of health performance evaluation, a major municipality can be considered to be one that has more than 100,000 inhabitants; b) Population data from the 2010 Demographic Census/Brazilian Institute of Geography and Statistics (Instituto Brasileiro de Geografia e Estatística - IBGE).

Most of the notifying services were hospitals (62.5%), in particular reference hospitals for comprehensive care for people in situations of sexual violence, which accounted for more than half (56%) of the notifications. The other notifying services were primary health care facilities (primary health care centers and Family Health Strategy centers), which accounted for 4.6% of notifications, and emergency care services, medical outpatient clinics, polyclinics and medical specialty centers, among other health and social services establishments, which accounted for 32.9% of the notifications.

## DISCUSSION

Notifications of sexual violence in Rio Grande do Sul, during the period studied, were more frequent among children and adolescents. There was a predominance of (i) pre-adolescent female victims and (ii) adult male aggressors, friends/acquaintances or relatives of the victims, for both race/skin color groups analyzed. Occurrences of sexual violence were more frequent at home, with co-occurrence of psychological/moral violence being most reported. We found higher prevalence and higher relative frequency of rape, sexual exploitation and co-occurrence of neglect/abandonment in victims of Black race/skin color; sexual harassment was more prevalent and frequent against people of White race/skin color. Considering the municipalities of Rio Grande do Sul individually, notifications were mainly concentrated in Porto Alegre; most of the other municipalities in the state had no or few notifications in the five-year period, with Alvorada and Pelotas standing out with the lowest case prevalence. More than half of the notifications were made by reference hospital services for people in situations of sexual violence, while in primary health care services recording of these occurrences was scarce.

Although the study was based on official notification data, it is possible that the results do not represent the actual frequency of the event because they included both confirmed cases and suspected cases of violence, which is a limitation. The SINAN data derives from the input of notification forms, filled in by health service professionals, which does not exclude the possibility of information bias. Another limitation of the study refers to the quality of the database, which was directly affected by the high percentage of variables not filled in or filled in as “unknown”, in addition to the exclusion of the “Asian” and “Indigenous” race/skin color categories and the grouping of the “Black” and “mixed race” categories into one, namely “Black”, resulting in only two categories in the study: i.e. “White” and “Black”. However, these populations that were grouped together or excluded deserve greater attention in research on sexual violence, making it important to conduct future studies targeting each of them, even though they are a small part of the general population.

The higher frequency of notifications of sexual violence against children and adolescents, found in the period analyzed, may indicate greater empathy and a tendency of the general community to report violence against vulnerable individuals. This finding possibly stems from the public policies implemented in the country in recent years. ^(^ ^4^ ^),(^ ^6^ A qualitative survey of the perception of primary health care workers regarding situations of domestic violence in a medium-sized municipality in Southern Brazil, in 2017, found that these professionals tend to rally, especially when violence is perpetrated against children and elderly women, for example.^15^ Even so, among the different types of violence that can affect children and adolescents, sexual violence has been one of the most reported, especially among girls.^16^

Other studies evaluating the records of sexual violence notified on the SINAN, in addition to police and security force incidents, in recent years, show that in Brazil the frequency of pre-adolescent female victims and victims in age groups corresponding to adolescence is higher.^1^ ^),(^ ^16^ ^)-(^ ^18^ Furthermore, the fact that these occurrences are more frequent in the victims’ homes, perpetrated by male adult aggressors, friends, acquaintances or family members, is also an expected finding. The same descriptive studies cited^1^ ^),(^ ^16^ ^)-(^ ^18^ ^)^ indicated that the victims are mostly raped by a man, either a relative or an acquaintance, within their own home, which makes it difficult to reveal what happened and contributes to its being underreported.

These results are related to cultural factors based on the dominant male identity, for which the conception of family is influenced by a patriarchal and male chauvinist (*machista*) model, which considers women and children as being the property of men.^19^ It is possible that these factors may have contributed to the low frequency of notifications of sexual violence in male children and adolescents found in our study, corroborating a theoretical essay on sexual violence against boys in Brazil, carried out in 2012.^20^

We found a considerable amount of co-occurrence of psychological/moral violence. A case series study that analyzed records of sexual violence and co-occurrences, collected from guardianship councils (*conselhos tutelares*) and social service reference centers (*centros de referência em assistência social* - CRAS) in the municipality of Feira de Santana, state of Bahia, beween 2001 and 2010, also found that psychological violence was the event most frequently associated with sexual violence.^21^ This factor, along with the others already mentioned, contributes to sexual abuse, which usually manifests itself when there is an asymmetrical relationship between the victim and the aggressor. One of the forms of asymmetry is the difference in age, the difference being five years in the case of a child under 12 years old, and ten years when the victim is over 12 years old. Another form of asymmetry is found in the power relationship in which the aggressor exercises some sort of control over the victim or uses emotional blackmail and psychological manipulation, which usually occurs when he is a man, head of the family or someone very close to the victim.^22^

The types of sexual violence we found manifested themselves differently according to race/skin color. Although there was higher prevalence of general notifications for victims of Black race/skin color, specificity of sexual harassment was more frequent among White victims, when compared to Black victims. We also found this difference for rape, sexual exploitation and co-occurrence with neglect/abandonment, these categories being more frequent among Black victims compared to White victims. One of the forms of sexual exploitation in Brazil relates to children and adolescents who, neglected by their families, start to live on the streets where they often resort to prostitution as a means of survival. This problem in Brazil, where society is strongly based on the social construction of the subalternization of Black bodies as consumer goods, ever since the time when slavery existed,^23^ possibly influences the greater number of rapes, prostitution and negligence found among Black people. Likewise, the higher number of occurrences of sexual harassment among White people appears to be conditioned to the stereotype of beauty socially constructed by whiteness,^24^ established in Brazil ever since European colonization.

With the exception of the state capital, Porto Alegre, which concentrated most of the notifications, most municipalities in Rio Grande do Sul had very low numbers of sexual violence; in particular Alvorada and Pelotas had the lowest prevalence rates in the five-year period analyzed. These numbers do not necessarily imply low frequency of sexual violence against children and adolescents. A descriptive study that assessed violent crime in Brazilian municipalities based on national data from the Mortality Information System (*Sistema de Informações sobre Mortalidade* - SIM) in 2015, identified Alvorada as the only municipality in the state of Rio Grande do Sul appearing in the ranking of the most violent municipalities in Brazil, and the municipality of Pelotas as one of those with the highest homicide rates.^25^ An ecological study, which analyzed the relationship between feminicides and socioeconomic indicators in Brazilian state capitals and large municipalities, in 2007, 2009, 2011 and 2013, showed that municipalities with greater urban violence had greater social and gender inequality, in addition to higher risk of occurrences of sexual violence.^26^

The main services notifying sexual violence in Rio Grande do Sul were reference hospitals for care for people in situations of violence, while the number of notifications made by primary health care services was low, possibly due to the shortage of staff trained to identify and report cases. This problem can contribute to greater vulnerability of victims and their families, who, in most cases, have to seek care in places that are more distant and harder to get to. A study analyzing the process of training civil servants to address sexual violence against children and adolescents in four Brazilian state capitals - including Porto Alegre - between 2010 and 2011, found little investment in training health professionals to prevent violence and promote protective bonds.^27^ Training on the subject was mostly focused on specialized health care,^27^ making it necessary to expand training to primary health care services, given that they are the entry point for users of the public health system, so as to make them more accessible for the community.

To sum up, our study found a higher frequency of notifications of sexual violence against females and the pre-adolescent age group, and a higher prevalence of notifications and relative frequency of rape, sexual harassment and sexual exploitation in victims of Black race/skin color (i.e. Black and mixed race). We recommend that protection measures aimed at these groups in health services be strengthened. The distribution of notification frequency was low in PHC services, pointing to the need to strengthen surveillance in these services, especially in municipalities with higher rates of violence.
