# Erratum

**DOI:** 10.1590/S1679-49742023000200027

**Published:** 2023-08-21

In the article **“Notifications of sexual violence against children and adolescents in Rio Grande do Sul, Brazil: a descriptive study, 2014-2018”, doi: 10.1590/S2237-96222023000200004**, published on Epidemiology and Health Services, 32(2):e2022853, 2023, in the page 01:

Original text:

Luciane Maria Piloto

Corrected text:

Luciane Maria Pilotto

